# Understanding the FDA Process Is Critical for Young Companies

**DOI:** 10.1089/jmedxr.2024.0053

**Published:** 2025-04-08

**Authors:** Jesse Courtier, Rick Beberman

**Affiliations:** ^1^ UCSF Benioff Children’s Hospital, San Francisco, California, USA.; ^2^ UCSF Department of Radiology and Biomedical Imaging, Sira Medical, San Francisco, California, USA.; ^3^ Sira Medical, San Francisco, California, USA.

**Keywords:** clearance, Food and Drug Administration, Food and Drug Administration consultant, regulatory, software development, startup

## Abstract

Our team recently led our augmented reality preoperative planning software application through Food and Drug Administration 510(k) clearance as a class 2 medical device. As augmented and virtual reality play an increasing role in health care, more early-stage companies will be required to seek this clearance. Often founders at these companies lack regulatory experience, which can make the process difficult and inefficient. Our experience was the first time for us. For those in a similar position, it’s important to keep a few things in mind that can help lead to a successful process.

## Introduction

Interest in applications of augmented reality (AR) and virtual reality (VR) from the leading technology companies is well known. Microsoft, Google, Apple, Samsung, Meta, and others have dedicated enormous resources to AR/VR development.^1–3^ With this kind of market sponsorship, entrepreneurs within health care, not surprisingly, see the possibility for startup success. Many of these entrepreneurial efforts, though, can have an impact on patient safety. As the regulatory body that oversees software applications that impact patient safety, the Food and Drug Administration (FDA) provides standards and actively assesses and clears these products. The FDA has, as of this past September, cleared 69 AR and VR products.^4,5^ If your application has an impact on patient safety, you will need to seek FDA clearance.

AR and VR have the potential to bring clear advantages to many areas of health care. Improving patient safety and comfort, making clinicians more effective, and delivering efficiencies are just a few of these potential advantages.^6^ Given FDA oversight, having a working understanding of the regulatory process at a startup is a clear advantage, not just in terms of commercial success, but ultimately in being able to deliver better health care.

Unfortunately, many startups lack regulatory expertise and enter the process without full understanding of the requirements for FDA submission. Understanding this process early on, especially with respect to software development, can aid startups in avoiding costly missteps, delays in product launch, and ultimately, potential failure. Prior to filing for FDA clearance, it is critical to receive significant feedback on your application from clinicians and other users, ideally through extensive pilot testing.

A number of excellent online resources and educational materials are available, providing detailed overviews of the FDA 510(k) clearance process and device classification determination including the FDA’s own website.^7^ The following considerations can also aid organizations navigating the FDA clearance process for the first time.

## External Consulting

**FIG. 1.**
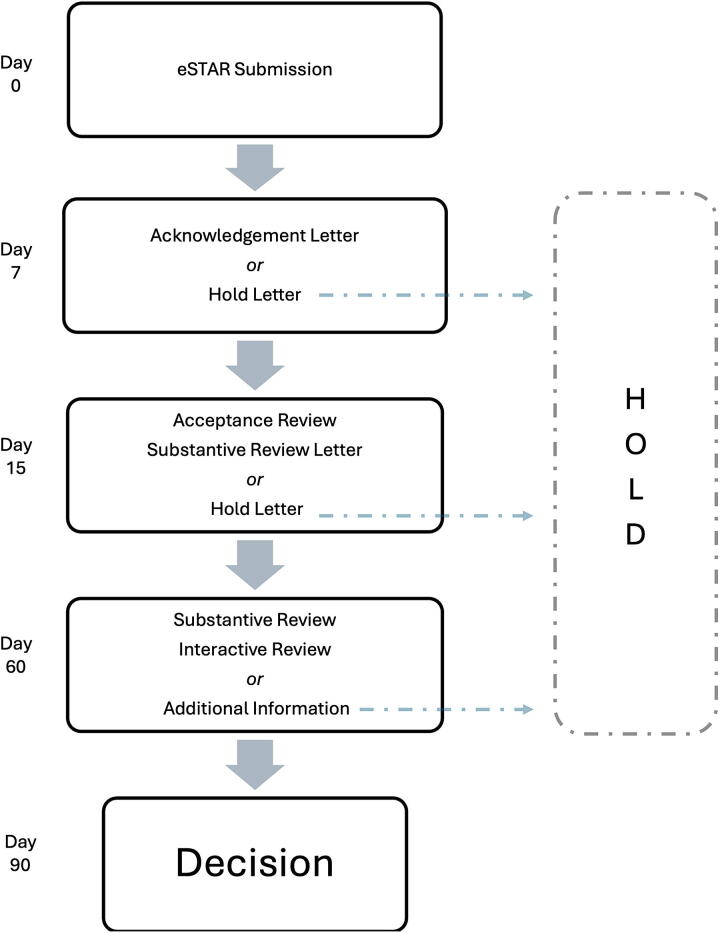
Order of events and timeline for a standard FDA 510(k) clearance process in the substantial equivalence pathway. FDA, Food and Drug Administration.

1. Professional consultants are a necessity—experts who are intimately familiar with the FDA and have gone through the process countless times. FDA consultants who understand software and ideally AR or VR should be sought out. This may appear to be obvious, but it is crucial for a smooth, efficient, and cost-effective process. Many of these consultants can be found in previous FDA filings. Budgeting for this part of the process is critical, and potential for added, unanticipated testing should be factored in.
2. While FDA 510(k) clearance is technically shown as being a 90 day process, there are a number of steps along the way where delays can occur (Fig. 1). Experienced consultants can provide guidance through any possible delays.

## Process Review and Improvement

1. Startups need to set up a formal quality management system, typically in conjunction with FDA consultants, to ensure:
  a. Continued compliance with regulations and standards
  b. Consistent quality processes across the company
  c. Accountability among team members to maintain quality and safety at the company
  d. Maintenance of audit trails and other effective record keeping

This is a time consuming and not inexpensive process.

2. Extensive review of all hazards users face while deploying the application and plans to mitigate these risks. Consideration for loss of functionality, file inaccessibility, or a user working from an incorrect plan must be made. These are just a few of the risks that must be identified and mitigated.

## Software Development

1. Critically review your software application and requirements. Are all features absolutely necessary to achieve your application’s primary mission? Extraneous software features can equate to a longer and more expensive process.
2. Software developers will play a critical role in clearance and will likely spend more time with FDA consultants than anyone else in the organization. Developers need to provide evidence of how the organization’s software was developed. This includes providing extensive documentation covering:
  a. Software requirements
  b. Design charts
  c. Software lifecycle development
  d. History of revisions and all anomalies including any unresolved ones
  e. Testing for validation
  f. Maintenance activities going forward

 Ensure your developers are aware of all that will be required of them at the earliest stage of software design. Developers play a critical role in a successful clearance process.

3. Cybersecurity is an FDA current topic of high interest. Extensive threat modeling, thorough descriptions of controls on hardware, software bills of materials, risk mitigation, and traceability matrices are just a few of the items that will need to be provided.

## External Testing

1. Clinical trials may not be necessary, but usability testing likely will be. Companies will be required to retain an independent testing firm to set up testing protocols and run 15+ clinicians through every element of the application to determine whether the clinicians can successfully complete assigned tasks. Many of them may be putting on a head-mounted display for the first time, and training will consist of them having read your user manual once or twice. It’s often best to keep the software simple and straightforward.
2. Depending on the nature of the software application, retaining an optical lab to measure the application’s visual performance with the head-mounted display may be necessary, particularly if there is a surgical planning aspect. Visual testing includes measuring luminance, contrast, and resolution among other elements to ensure users can use an application and clearly see three-dimensional images, including under different environmental conditions.

## Seek Guidance

1. Founders should connect with as many people as possible who have successfully maneuvered AR/VR software through the FDA about how long preparation for filing and the filing process take as well as what it will cost. In this way, time and expenses can be effectively budgeted. Continued engagement with users as changes are made to the application to ensure utility. Also, remember the FDA does not permit product marketing prior to clearance. So, it’s best to understand the journey as early as possible within the product lifecycle to manage relationships with your employees, vendors, potential customers, and funding sources.

 Finally, AR/VR in health care is still nascent, and FDA requirements will vary from one application to the next. A key question to address is if a similar, currently FDA-cleared product exists (substantial equivalence pathway) or not (*de novo* pathway). Further, as the software penetrates health care and evolves, and as risks become more evident, FDA requirements for clearance during the process can change. Adaptability is paramount—these requirements can indeed shift during the process.

## Abbreviations Used

AR: Augmented reality
FDA: Food and Drug Administration
VR: Virtual reality
